# Percutaneous CT guided radiofrequency thermoablation versus Magnetic Resonance guided Focalized Ultrasound Surgery (MrgFUS) in the treatment of Osteoid Osteoma. Critical evaluation of the indications

**DOI:** 10.1186/2050-5736-2-S1-A9

**Published:** 2014-12-10

**Authors:** Armando Conchiglia, Francesco Arrigoni, Gregori Lorenzo Maria, Luigi Zugaro, Carlo Masciocchi

**Affiliations:** 1 University of L'Aquila Department of Radiological Sciences, L'Aquila, Italy

## Background

MRgFUS represent a new therapeutic option for the non-surgical management of osteoid osteoma. The site of the lesion is the most significant limitation of use of this technique.

Purpose of this study is to compare the clinical and morphological two years after the procedure, of the treatment of 28 osteoid osteomas with Magnetic Resonance guided Focus Ultrasound Surgery (MRgFUS) versus the treatment with Radiofrequency termoablation (RF).

## Methods and materials

From March 2011 we treated 28 osteoid osteomas, 13 using MRgFUS (ExAblate InSightech, Israel) and 15 using RF (LeVeen Needle Electrode Boston Scientific - USA). The osteoid osteomas treated with MRgFUS were located in the femour (n.9), tibia (n.3) and in the talus (n. 1). The lesions treated with RFs were located in the femour (n. 9), talus (n.2), vertebral body (L3 and L5) and tibial plateau (n.2). All the lesions were diagnosed by plain films, CT and MRI and controlled after the procedure by MRI and CT. The clinical evaluation was performed by VAS scale.

## Results

All the patients but one treated with RF termoablation showed a regression in painful symptomatology with a mean VAS decreasing from 8 to 1.2 two years after the treatment. The treatment with MRgFUS was successful in 11 out of 13 patients (mean VAS dropped from 8.1 to 1.3 two years after the treatment). The two cases unresponsive were re-treated successfully with RF. The MRI evaluation showed a disappearance of bone edema already to the first controls at 6 months after the treatment in all the patients treated successfully. In the CT controls no substantial changes were found, except for the disappearance of the central calcification of the nidus in the 40% of cases treated with MRgFUS.

## Conclusion

Although further studies with a longer term and a larger number of cases are needed, our experience demonstrates the effectiveness of the treatment of osteoid osteomas with MRgFUS. In particular this treatment is successful in the 84,6% of cases. The main limit is today represented by the accessibility of the lesion by the ultrasound. However the treatment is repeatable and does not preclude treatments with other techniques.

